# Bee pollination and bee decline: A study about university students’ Knowledge and its educational implication

**DOI:** 10.1093/biosci/biae099

**Published:** 2024-10-26

**Authors:** Paula Daza, Montserrat Arista, Regina Berjano, Pedro Ortiz, Hortensia Morón-Monge, Yasmine Antonini

**Affiliations:** Universidad de Sevilla, Facultad de Biología/Facultad de Ciencias de la Educación, Departamento de Biología celular, Sevilla, Spain; Universidad de Sevilla, Facultad de Biología, Departamento de Biología vegetal y Ecología, Sevilla, Spain; Universidad de Sevilla, Facultad de Biología, Departamento de Biología vegetal y Ecología, Sevilla, Spain; Universidad de Sevilla, Facultad de Biología, Departamento de Biología vegetal y Ecología, Sevilla, Spain; Facultad de Ciencias de la Educación, Departamento de Didáctica de las Ciencias experimentales y sociales, Sevilla, Spain; ICEB-Universidade Federal de Ouro Preto, Departamento de Biodiversidade e Evolução, Ouro Preto, Brazil

**Keywords:** education, plant–animal interaction, environmental science

## Abstract

Science education plays a crucial role in addressing the pollinator crisis by enhancing knowledge and fostering changes in attitudes toward this environmental challenge. Previous research has been focused on validating a specific instrument related to this subject, although its use for assessing students’ knowledge has been little explored. In the present study, we have evaluated the level of awareness regarding the significance of bees as primary pollinators among students of various disciplines at the Universities of Sevilla, in Spain, and Ouro Preto, in Brazil, emphasizing the importance of the plant–bee interaction. 753 students from the fields of biology, agriculture, and education were invited to complete a questionnaire focused on bee biology. The results indicate that knowledge on the subject is closely linked to professional career choice and that the training program for the future teachers effectively increased comprehension of the crucial role played by bees as main supporters of the ecosystem service of pollination.

Pollination in angiosperms, the transport of pollen from the anthers to the stigma, is a vital process in the reproductive cycle of plants because it ensures fruit and seed production. Pollen transport can be carried out by abiotic vectors such as wind or water or biotic vectors such as animals. Most wild plants (87%) are pollinated by animals (Ollerton et al. 2011) through mutualistic interactions that are fundamental for the conservation of both the plants and the animals that interact with them, making pollinator–plant interactions vital for the maintenance of biodiversity (Potts et al. 2023). In fact, a positive correlation has been found between plant and pollinator diversity (Heithaus 1974, Del Moral and Standley 1979). Not only are pollinators crucial for the reproduction of many wild plant species, but many economically important crop species also heavily rely on pollinators to produce fruit and seeds (Hung et al. 2018, Lindao-Cordova et al. 2020, Durazzo et al. 2021). Although certain crops, particularly cereals, are wind pollinated and although agricultural selection has rendered some independence of pollinators through parthenogenesis, approximately three-quarters of all crop species are reliant on animals—predominantly insects—to produce high-quality fruits (Klein et al. 2007, Khalifa et al. 2021). The proportion of crops dependent on animals has seen a substantial increase in the past 50 years (Aizen et al. 2008, Potts et al. 2010, 2016, Goulson et al. 2015, Koh et al. 2016). Consequently, solely from an economic perspective, preserving robust pollinator populations should be prioritized in food policy to ensure the safety of food systems both nationally and internationally (Murphy et al. 2022).

On the other hand, pollination is considered an ecosystem service—that is, an ecological function critical for human survival—and this service is mediated to a large extent by bees (Klein et al. 2018). Bees are considered the most important pollinators worldwide of both wild and cultivated plants (Klein et al. 2007, Brittain et al. 2013a, 2013b, Satyshur et al. 2023). The significance lies in the fact that bees rely entirely on floral rewards for their sustenance, which leads to their high level of activity as frequent visitors to flowers. In addition, because bee’s bodies are covered with hairs, much of the pollen sticks to them when they forage on the flowers, and it is available for pollination, making them highly efficient pollinators (Potts et al. 2016). About 20,000 bee species have been described, although their number will probably be higher because it is considered that many species have not been described yet (Orr et al. 2021). It can therefore be affirmed that the diversity of bee communities ensures a high and stable provision of pollination services (Hoehn et al. 2008).

Many studies in the past two decades have warned about pollinator decline. A global meta-analysis revealed a 45% decline in insect abundance (Wagner 2020). Bee decline may be particularly troublesome because of the potential effects on plant reproduction; in fact, there is evidence that bee pollinators and wild plants are linked and declining in parallel. Among the main causes of pollinator decline, intensive agriculture is recognized as one of the most important and increasing threat to bees and their ecosystem services (Kremen et al. 2002, Attwood et al. 2008, Kremen and Miles 2012, Goulson et al. 2015, Grab et al. 2019, Nottebrok et al. 2022). The transition from natural to agricultural lands is a primary driver of biodiversity loss worldwide. On the other hand, other commonly reported threats include pathogens, pesticides, invasive species, and climate change (Vilá et al. 2011, Vanbergen et al. 2013, Goulson et al. 2015, Wagner 2020).

In light of compelling evidence detailing the decline of bees and its far-reaching impact on biodiversity and human health, significant resources, research, and initiatives have been dedicated to addressing the dwindling bee populations. Despite these efforts, little substantial progress has been observed over the past few decades. In this context, education can play a crucial role in communicating the need to conserve pollinators to preserve human well-being, and it is a key tool that can effectively lead to behavioral changes in citizens enhancing engagement in science learning (Saunders et al. 2020, Satyshur et al. 2023) and ultimately fostering environmental citizenship (Hadjichambis and Reis 2020), as well as action competence in sustainable development (Sass et al. 2020). Therefore, it is crucial to train preservice primary teachers (education students) who convey this necessity. Numerous studies have established a positive correlation between higher education and environmental concern (Torgler and García-Valiñas 2007). This correlation is causally attributed, because individuals with higher education levels typically exhibit a greater proclivity toward environmentally friendly behaviors (Meyer 2015). Therefore, education could increase knowledge encouraging changes in attitudes toward environmental problems. From an environmental citizenship perspective, awareness about pollinators can be promoted (Cho and Lee 2018) in order to boost active and responsible citizenship to get involved in the decision-making processes concerning controversial socioscientific and sociotechnical issues (Hadjichambis and Reis 2020).

To our knowledge, only a handful of studies have delved into the public's understanding of pollination and pollinators (Golick et al. 2018, Hall and Martins 2020, Jimenez et al. 2022, Vlasák-Drücker et al. 2022). We think that universities are key instrumental in preparing people for important social roles (Frank and Meyer 2007), and therefore, university students with knowledge of environmental problems could be leaders in the development of positive attitudes toward the environmental concern for pollinators that are in decline (Zilahy and Huisingh 2009, Lozano et al. 2013). This could be especially important in those areas more related to environmental conservation or where the pollinator crisis is especially relevant, such as biology or agriculture. In addition, there is a group of students whose knowledge of environmental issues could have a much greater social impact. This is the case for the preservice primary teachers, because they will have the role of transmitting the information to the youngest levels of society, and who, in turn, will be responsible for the conservation of the environment in the future (Sotero et al. 2020, Ricoy and Sánchez-Martínez 2022). Insects are part of the living beings addressed in the subject of natural science in primary education in Spanish schools: First, children are taught to identify living beings and insects are included as a group of invertebrates. Later, students must learn main parts of the body of invertebrates, being the insects a very important group. Finally, scholars between 10 and 12 years old learn the animal body structures, but insects are not so relevant at this stage.

According to Gómez-Prado and colleagues (2022), preservice primary teachers should be affectively trained to avoid negative attitudes toward insects, and this could be directly related to learning significantly about the decline of pollinators. In addition, this socioenvironmental problem used as the thread of the subject allows contextualizing knowledge, promoting meaningful learning in our students (Moron-Monge et al. 2020). Although specific studies such as that of Jimenez and colleagues (2022) focus on validating a specific instrument to know the level of literacy on this topic, its application for assessing students’ knowledge levels has been less commonly pursued.

The aim of the present work is to explore the knowledge level about the relevance of bees as the most important pollinators of wild and cultivated plants (Willians 2002, Ollerton 2017) in a group of students at the University of Seville, in Spain, (USevilla) and the Federal University of Ouro Preto, in Brazil (UOuro). Furthermore, we aim to explore to what extent this knowledge about bees has evolved after an educational intervention with preservice primary teachers. Many of these students will develop their professional activity in areas where the pollinator crisis could have a particularly important impact because of, first, its markedly high plant and bee diversity (Mitermeier et al. 1997, Myers et al. 2000, Michener 2007, Ascher and Pickering 2020) and, second, agriculture as one of the most important economic activities in these countries. Therefore, we have focused our study on students of agriculture and biology because of the curricula of these programs are related to environment conservation or plant pollination. Moreover, because of the multiplier effect of the teachers on society, we have also assessed the level of knowledge about bees on the preservice primary teachers, because of its role as future educators. It would be expected that preservice primary teachers would show less knowledge about bees and pollination than biology and agriculture students; however, given their role as early childhood educators, they could have a greater future impact on concern about these aspects.

## Research setting and design

USevilla was founded in 1551, and it is the third largest Spanish university in terms of the number of student and the diversity of disciplines. It has 69,676 alumni between undergraduates and postgraduates, 4395 professors, and 2860 administration staff members. UOuro was created on 21 August 1969, with the merger of the century-old and traditional School of Pharmacy and School of Mines. It has 13,339 alumni between undergraduates and postgraduates, 1927 professors, and 663 administration staff members. The research team is composed of people from the two different universities, five from USevilla and one from UOuro. Most of us give lessons in biology, some of us also teach in the agriculture faculty, and two of us are professors of primary education in the education faculty.

Students from the disciplines of biology, agriculture, and education (preservice primary teachers) were selected for the following reasons: First, biologists are directly implicated in environmental and nature conservation, and they are the main researchers in biodiversity; second, agronomist are directly implicated in crop production and worldwide feeding; finally, preservice primary teachers are the main group responsible for transmitting the information to the youngest levels of society, who, in turn, will be responsible for the conservation of the environment in the future. In addition, we included biology students from two different countries considered biodiversity hotspots (Spain and Brazil), in which education curricula and concern for biodiversity conservation differ.

In the present study, we designed an initial questionnaire with a total of 20 questions based on both the literature (Klein et al. 2007, Potts et al. 2010, Ollerton et al. 2011, Cho and Lee 2018, Golick et al. 2018, Grab et al. 2019, Knapp et al. 2020, Patel et al. 2021, Jimenez et al. 2022, Ojija and Leweri 2022) and the knowledge of the study authors who are experts in plant reproduction and education (a panel of experts). The initial questionnaire was administered to 35 students enrolled in the biology degree as a pilot test, aimed at assessing the questionnaire's validity and refining it on the basis of the obtained results. After this revision, some questions were reformulated, and others were eliminated because they were confusing. As a result, we achieved a questionnaire composed of 16 closed questions, including 11 multiple choice questions with only one possible answer, four true–false questions, and one ranking question based on a Likert scale (supplemental table S1).

All of them addressed three dimensions or categories of analysis related to our research topic (table 1). The establishment of analysis categories is a common analysis strategy in science education to explore knowledge (Porlán et al. 1997, Adb-El Khalick and Lederman 2000, Acevedo-Díaz and Acevedo-Romero 2002). In our study, focused in particular on the knowledge about bee pollination and bee decline, the dimensions of analysis have been established on the basis of previous studies as seen in table 1.

**Table 1.** Questionnaire and analysis dimensions.

Dimensions		Descriptors	Questions	Reference of the dimension
Knowledge about pollination	Conceptualization and relevance from the ecological point of view	Conceptualization, function, and relevance from the ecological point of view	Q1, **Q3** and Q4	Knapp et al. 2020, Golick et al. 2018, Jimenez et al. 2022
	Relevance from the human point of view	Function and relevance of the pollination from the human point of view for their horticultural and economic activities	Q2, **Q11**, Q12 and Q15	
Knowledge about bees		Types of bees and biodiversity, function, and ecological relevance	**Q5**, Q6, **Q7**, Q8, **Q9** and **Q10**	Cho and Lee 2018
Environmental perceptions and sources of knowledge		Sources of knowledge about the problem of loss of pollinators’ biodiversity and its environmental perception	Q13, Q14 and **Q16**	Knapp et al. 2020, Ojija and Leweri 2022

*Note:* The questions marked in bold were selected for further analysis. The selection was made on the basis of the differences found.

The validation of the teaching protocol has been performed using specific literature and the expertise of the panel of experts (table S1), as it has been previously performed by various authors (Cho and Lee 2017, Knapp et al. 2020, Ojija and Leweri 2022, Vlasák-Drücker et al. 2022). The final version of the validated questionnaire was distributed to the selected students, a total of 704 Spanish students from three different disciplines: biology (*n* = 152), agriculture (*n* = 49), and preservice primary education (*n* = 503), as well as 49 Brazilian students from the biology degree. The students were asked to fill out the questionnaire with the 16 issues related to bee biology and pollination. The questionnaire was provided to all of the students enrolled in the selected courses at the beginning of the academic year. Prior to the data collection, a set of ethical principles was carefully considered. The participants were regarded as competent research subjects, possessing the capacity to provide informed consent or decline participation in the study. The researchers maintained a comprehensive understanding of the participants’ requirements and progression, collaborating closely with classroom educators throughout the process. In total, the data collection process entailed approximately 1 hour per degree program. Disparities in enrollment numbers between male and female students in these courses led to a gender bias, resulting in an imbalance in gender distribution across the programs, with the exception of the agriculture students, where male and female students were proportionally represented.

### Specific protocol designed for preservice primary education

In order to promote the knowledge of preservice primary teachers about the importance of bees and pollination for human beings, a teaching protocol focused on this topic and based on project-based learning was designed. This protocol was developed throughout the academic year for these students (*n* = 503; 404 women and 95 men), within the life sciences subject of the second semester, with a total of 45 hours (35 theoretical session hours and 10 laboratory and field practical hours). For that, three main teaching actions were carried out that make up the teaching protocol: adoption of specific contents and activities related to bee biology to be transversally worked on throughout the subject, seminars given by bee experts, and scholar research on bees developed by the students. This last activity of the protocol is the final product of the students in which they used research strategies to finally present their results in a poster format. The students filled out the questionnaire at the beginning of the semester (pretest), and, at the end of the course, they filled out the questionnaire again (posttest) so that we could compare the knowledge acquired from the teaching protocol.

Table 2 shows the adaptation of the contents of the life sciences subject to the bee and pollination matter. The subject is made up of six thematic blocks divided into 10 topics, ranging from aspects more related to life molecules (microcosm) to more complex issues such as ecosystem relationships (macrocosm), going through more specific issues on the biology of plants and animals, as well as related to evolution and heredity (mesocosm), organized from a perspective that allows the construction of complex knowledge (Rodríguez-Marín and García-Díaz 2009). Moreover, we cared about the correspondence between the contents worked on in teacher training and that included in the primary education curriculum in Spain (Ministerio de Educación de España 2022). As can be seen, in addition to the issues that Gómez-Prado and colleagues (2022) pointed out, specific to insects (mainly morphology and classification), and within the primary education curriculum, other aspects related to the importance of these living beings and their interactions with plants and animals were afforded, as well as their importance for the human being and its economy.

**Table 2.** Different topics extended during the development of the subject life sciences.

Teacher training			Primary school
Subject life sciences		Adaptation: bee and pollination	
Thematic blocks	Topics	Contents	Curriculum natural science in primary education
Block I. Unit of Life	BiomoleculesThe CellMetabolism	Honey is a Sugar Wax is a Lipid Royalactin is a ProteinDNA and Demethylated Queen genes Royal jelly and EpigeneticsBee Bread and Lactic Fermentation	Observable properties of materials, their origin, and their use in everyday objects in accordance with the design needs for which they were manufactured Pure substances and mixtures Identification of homogeneous and heterogeneous mixtures Separation of heterogeneous mixtures by different methods
Block II. Principles of inheritance	Cell Cycle	Haplodiploidy: Parthenogenesis and Fertilization	
Block III. Evolution and Diversity of life	Evolution and Biodiversity	Coevolution of Bees and Flowers	Relationships between humans, animals, and plants
Block IV. Plant Biology	Plant Structures	Androecium and Gynoecium: Pollination	
	Plant Classification	Angiosperms and Flowers	
Block V. Animal Biology	Animal Structures	Royalactin or Regina and Stem cells	Main parts of the body of invertebrates (being the insects a very important group)
	Animal Classification	Metamorphosis of Arthropods: Egg, Larva, Pupa and Adult	Characteristics of the living beings (insects are included as a group of invertebrates)
Block VI. Ecology	Ecology	Importance of the Bees for the Biodiversity, Farming and Human beings	The animal body structures (insects are not so relevant at this stage) Relationships between humans, animals, and plants. Care and respect for living beings and the environment in which they live, preventing the degradation of soil, air, or water

Not only was the life sciences subject adapted, but another of the actions carried out was the organization of a seminar with an expert. This conference discussed the importance of bees as a new concept for the students, such as their relevance for ecosystem services. Furthermore, during and after the seminar, the preservice primary teachers engaged in discussions regarding alternative ideas, myths, or prevalent misconceptions surrounding the biodiversity of bee species and their significance in biodiversity conservation and the economy. These discussions fostered active participation among the students, who diligently took notes for subsequent use in a collective classroom presentation and in preparation for their individual school investigations.

In the third teaching action, as a product of learning, the students carried out school research in work groups of three or four students on some of the topics worked on throughout the course (table 3).

**Table 3.** Topics investigated (posters) and their correspondence with the content blocks of the subject.

	
Main topic posters	Blocks
	
Origin and evolution of the bees	Block III. Evolution and Diversity of life
Types of bees	Block V. Animal Biology and Block II. Principles of inheritance
Bee anatomy	
Bee life cycle: Metamorphosis	
Pollination	Block IV. Plant Biology and Block VI. Ecology
Plant and animal interaction: Mutualistic symbiosis	
Honeybees	Block I. Unit of life and Block VI. Ecology
Bee keeping	Block VI. Ecology
Bee dancing	Block V. Animal Biology
Hives collapse	
Apitherapy	Block VI. Ecology
Importance of the insect hotels placing	
Relevance of the bees for the planet: Biodiversity maintenance	
Relevance of the bees for the planet: Farming and economy	

**Figure 1.**
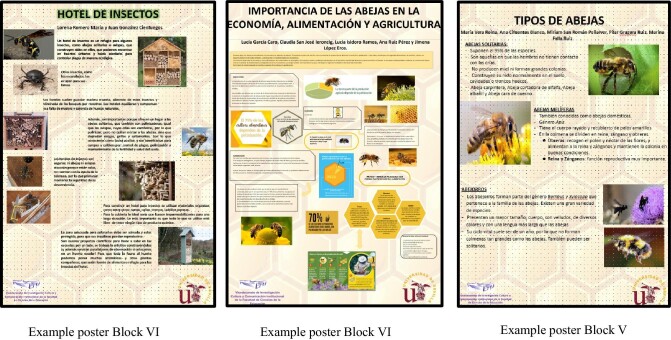
Example of posters produced by preservice primary teachers to present the most outstanding results and therefore disseminate and share them with the rest of the students.

In total, the students investigated 14 major topics (table 3) that correspond to the different blocks of content of the subject. These works were coordinated by the responsible teachers during their preparation to finally present the most outstanding results in poster format and therefore disseminate and share it with the rest of the students. Figure 1 shows three examples of the designed posters, two related to block VI and one to block V. Because this group research task was part of the final grade for the subject, there was a great involvement of the students throughout the process. This can also be observed in the qualifications they obtained, because they were among the activities carried out throughout the course that obtained the best score.

Other activities that were carried out during the practice hours and that were also interesting for the students were the insect hotel setting to observe these animals directly, the visits to the school garden of the university where the relationships of insects with plants could be supported, and the use of insect life cycle models to learn metamorphosis.

**Figure 2.**
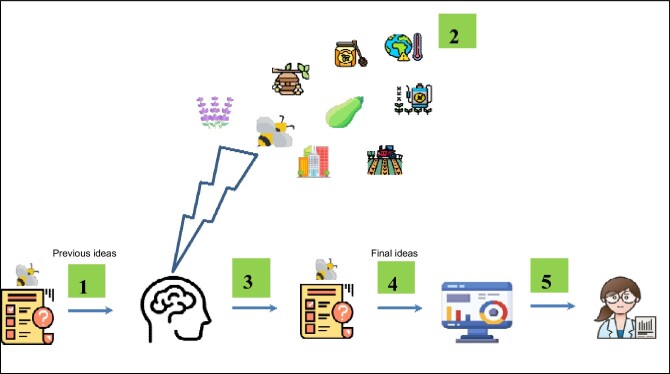
Conceptual diagram illustrating the process of specific protocol for preservice primary teachers: (1) data collection from the pretest about bee knowledge; (2) learning from project-based learning through the academic course; (3) data collection again from the posttest about bee knowledge; (4) data analysis and (5) reporting results obtained. This figure was designed with Flaticon.com resources (www.flaticon.com).

Finally, at the end of the course, the students filled out the questionnaire again (posttest) to compare the knowledge acquired from the teaching protocol during the semester. Summarizing, the most outstanding results on the learning generated after the experience are shown in the following section (figure 2).

### Data analyses

The answers across various student groups were analyzed using chi-square tests of frequency distribution to ascertain whether observed responses exhibited significant disparities among different disciplines: We examined the answers for each item among biology, agriculture, and preservice primary education; we compared the biology students’ responses from both USevilla and UOuro, we compared the responses of the preservice primary teachers before and after activities (pre- and postquestionnaire data; see item 2.2), and we compared the postactivity responses of preservice primary teachers with those of biology students to gauge knowledge improvement relative to the baseline knowledge of biologists, as a reference group, all of them from six selected questions (table 4). We chose these six questions because we found relevant the differences found among the three disciplines selected and because the other questions were invariant among the groups. The analyses were performed using the chisq.test function in R (R Core Team 2023). Post hoc tests following chi-square analysis were conducted using Bonferroni adjustment tests. The details of answers and analysis are available in supplemental tables S2, S3, and S4.

**Table 4.** Selected questions from the questionnaire to be analyzed.

Question	Answers (choices)	Dimensions
Do you know how most of the flowers are pollinated?	By wind By water By animals By humans	I
How many species of bees do you know?	Only one 2 to 5 6 to 10 More than ten	I
Do you believe that most bees are eusocial with queen, drones and workers?	Yes No I don't know	II
What do bees use pollen for?	To feed larvae To produce honey To build nests I don't know	II
Which of these is not a bee product?	Royal jelly Honey Wax Nectar	II
Which of these bees is the most efficient pollinator for tomatoes, peppers and eggplants grown in greenhouses?	Honeybee Solitary bee Bumblebee I don't know	I
Rank in order of importance the main reasons for the disappearance of pollinators, with 1 being the most important reason and 4 being the least important	Separate Urbanization and Diseases Modern agriculture Climate change Urbanization Diseases	III

*Note:* Selection was made on the basis of the differences found.

## Bee relevance for our university students

The selected questions inquired about pollination and bee knowledge (dimensions I and II) and about beliefs about the main causes of bee decline (dimension III; table 1)

### Pollination and pollinators’ knowledge

**Figure 3.**
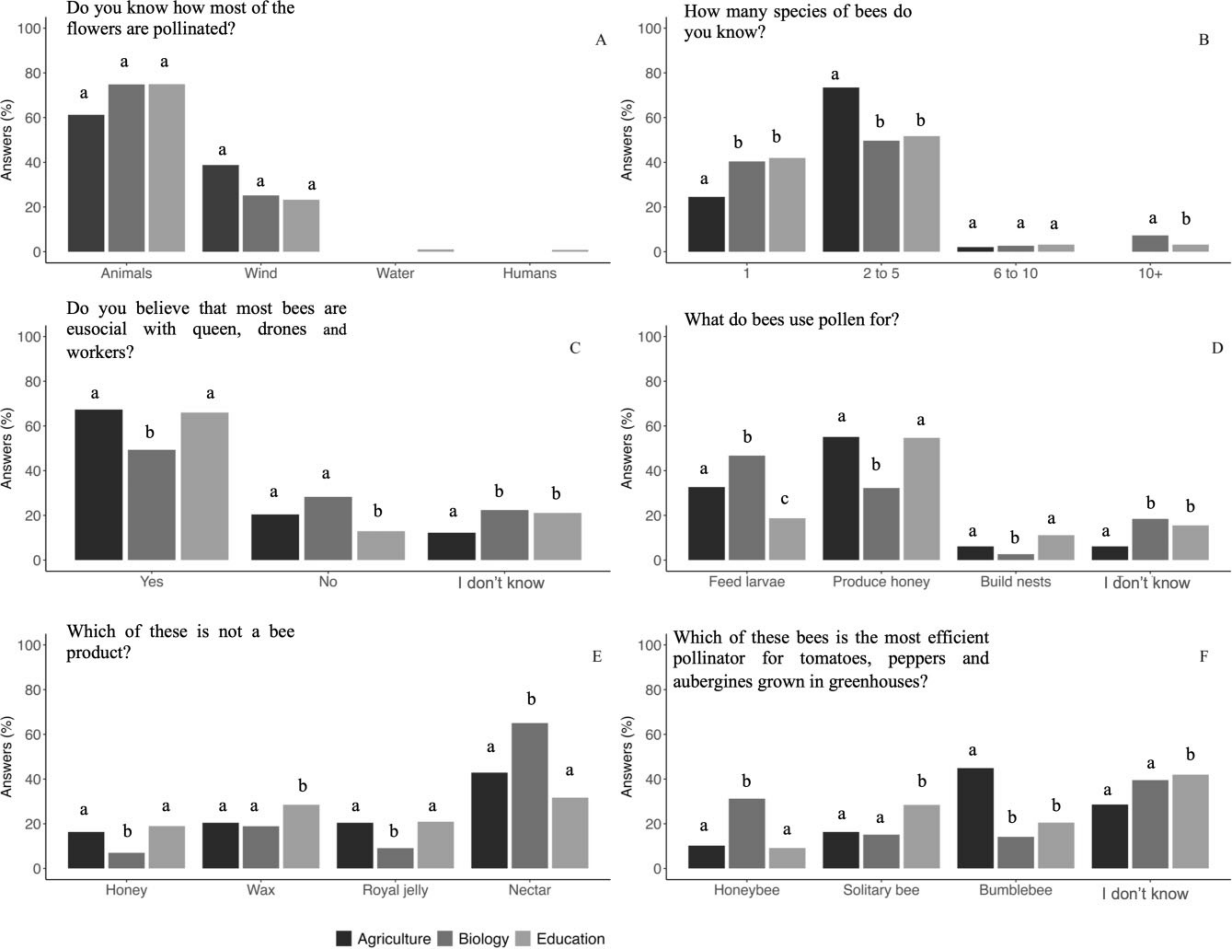
Knowledge of the concept of pollination, the most important pollinators, and the most efficient group of pollinators for Spanish students. For each answer, different letters among the columns represent significant differences among university degrees.

**Figure 4.**
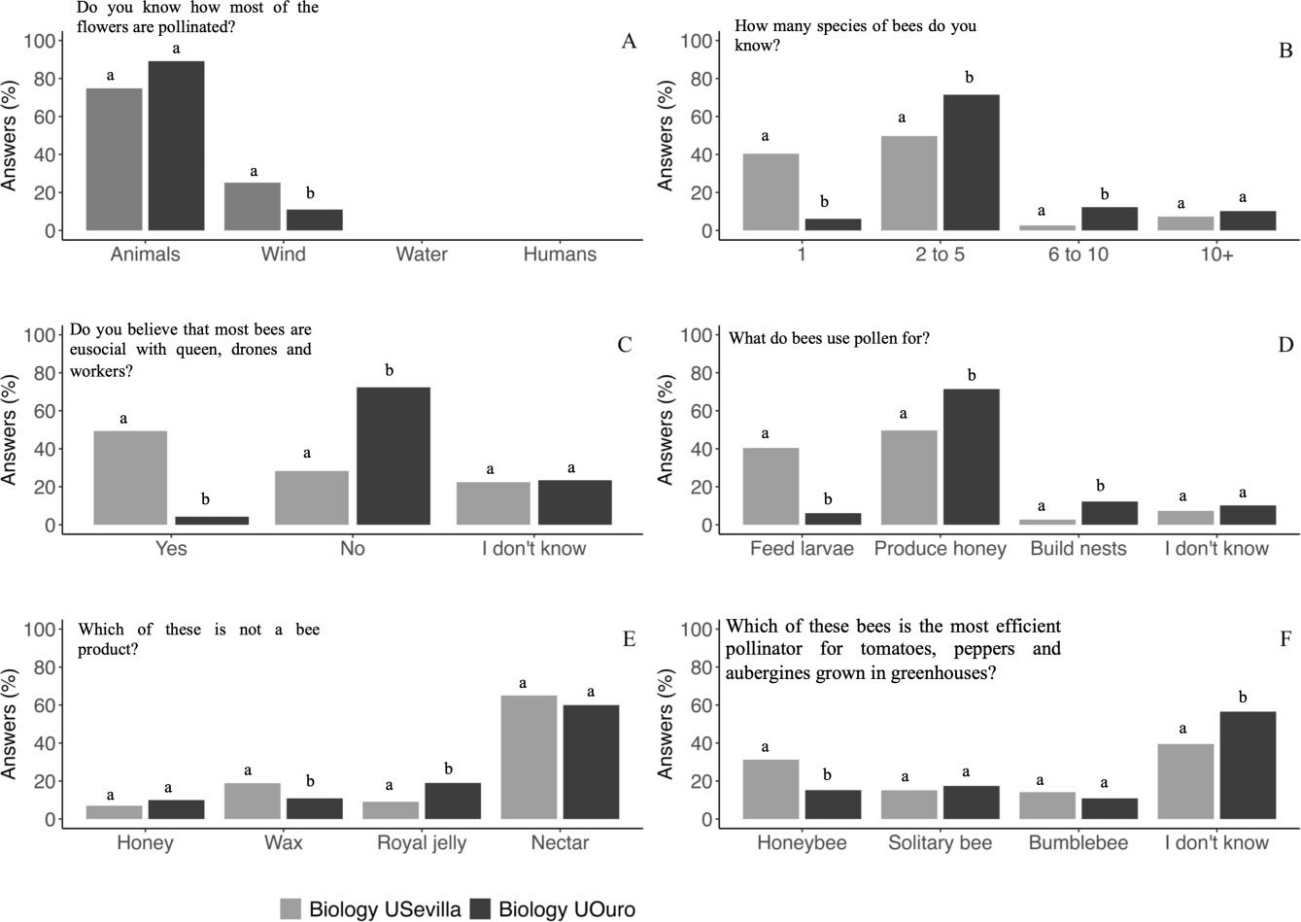
Comparative of the pollination concept knowledge, the most important pollinators, and the most efficient group of pollinators between USevilla and UOuro biology degrees. For each answer, different letters among the columns show significant differences between university degrees.

In general, students of the three disciplines demonstrated a solid and similar understanding of the pollination concept, including knowledge about both the primary pollinators and the most efficient pollinator groups. Across all disciplines (including students from USevilla and UOuro), there was a unanimous recognition of animals as the predominant pollinators, and bees were consistently identified as the most crucial pollinators by most of the students. However, a slightly higher but not significant percentage of the agriculture students (38%) believed that wind is the primary pollinator of most plant species (figure 3a). Although both groups identified animals as the most important pollinators, a significantly higher percentage of USevilla students identified wind as the most important pollinator vector (figure 4a).

Overall, there was a notable lack of knowledge regarding bee diversity among the students, with the majority indicating familiarity with no more than five bee species and between 20% and 40% reporting knowledge of just one species (figure 3b). Specifically, about 73% of the agriculture students asserted awareness of up to five distinct bee species, a percentage significantly higher than that reported by biology students or preservice primary teachers. When comparing only biology students from the two universities, 71% of the UOuro students indicated familiarity with two to five bee species, a significantly higher percentage than the biology students from the USevilla (figure 4b). There was no clear pattern observed in students’ knowledge regarding the number of existing bee species, although a majority perceived there to be fewer than 10,000 bee species (data not shown). Most students believed the actual number ranged from about 500 to 1000 species (66% among biology students; data not shown). Only a minority of the students suggested that more than 20,000 bee species exist, 17% in biology, 11% in preservice primary teachers, and 8% in the agriculture (data not shown). Between 12% and 22% of the students across various disciplines indicated a lack of awareness of the eusocial or solitary nature of most bees. Moreover, over 50% of students held the misconception that all bees exhibit eusocial behavior, with this percentage being notably higher for the preservice primary teachers and the agriculture students (around 67%) than for the biology students (figure 3c, table S3). Notably, this misconception was significantly more prevalent (75%) among the biology students at USevilla than among those at UOuro (figure 4c).

A notable proportion of the students across all disciplines (ranging from 50% to 67%) were aware that not all bees produce honey (data not shown). However, approximately 55% of the preservice primary teachers and the agriculture students incorrectly believed that honey is produced using flower pollen. Regarding the significance of pollen for larval nutrition, the biology students demonstrated higher awareness than did the other two student populations (figure 3d, table S3). However, this knowledge was not similar between the biology students at USevilla and those at UOuro (figure 4d), with a higher proportion (40%) of the USevilla students than the UOuro students (less than 15%) having correctly identified that pollen is used to feed larvae. Notably, a significant proportion of the agriculture students (47%) and the preservice primary teachers (32%), as well as a subset of the biology students (65%), correctly identified that nectar is not produced by bees (figure 3e). In this same direction, the biology students at UOuro correctly identified that nectar is not produced by bees as did a similar percentage of the biology students at USevilla (figure 4e).

When asked about the primary pollinators in greenhouse crops such as tomatoes, peppers, and eggplant, around 45% of the agriculture students identified bumblebees as the main pollinator, whereas 29% admitted to not knowing the answer (figure 3f). In contrast, the percentage of students unfamiliar with the answer was notably higher among the biology students and the preservice primary teachers (figure 3f, table S3). Moreover, this percentage was significantly higher among the biology students from USevilla than among those from UOuro (figure 4f). Also, around 31% of the biology students from the USevilla identified honeybees as the main pollinator, compared with 15% of the biology students at UOuro (figure 4f). For detailed results of the chi-square tests across disciplines in the USevilla, see table S3.

### Beliefs about the main causes of pollinator decline

**Figure 5.**
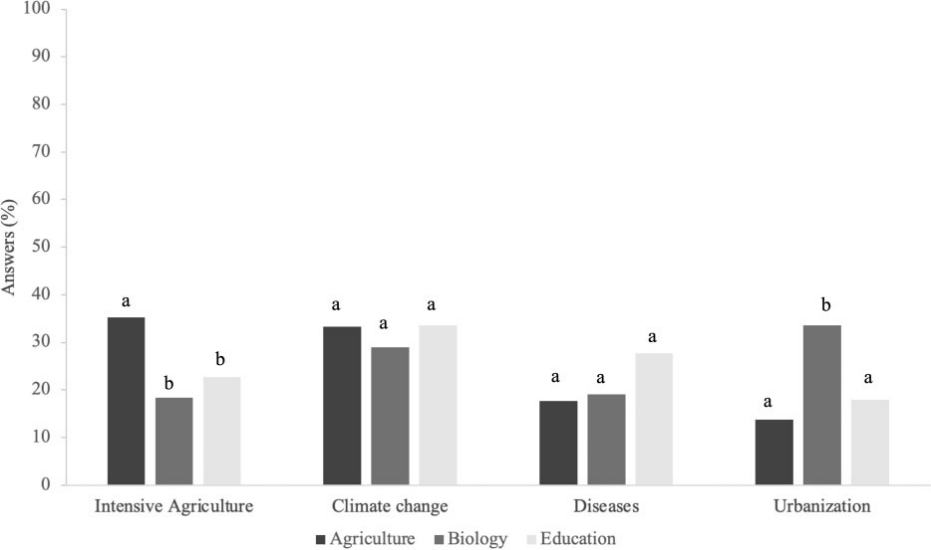
The perception of biology, agriculture, and preservice primary teachers at the University of Sevilla on the main cause of pollinator decline. Within each university degree, the different letters represent significant differences among the answers.

There were markedly different ideas about the causes of pollinator decline among the students from the three USevilla disciplines (figure 5). The percentage of the students attributing the decline of pollinators to climate change was approximately 30%, with statistically similar responses across disciplines. However, significant disparities emerged when considering other causal factors. Specifically, the biology students exhibited a notably high inclination toward urbanization as a main contributing factor in pollinator decline, significantly surpassing the students from agriculture and the preservice primary teachers in this regard (figure 5). Similarly, a considerable proportion of the agriculture students identified intensive agriculture as a leading cause, with this percentage being significantly higher than that for the biology students and the preservice primary teachers, who attributed the diseases as a main cause of pollinator decline (figure 5).

### Perception about the importance of pollination of preservice primary teachers

**Figure 6.**
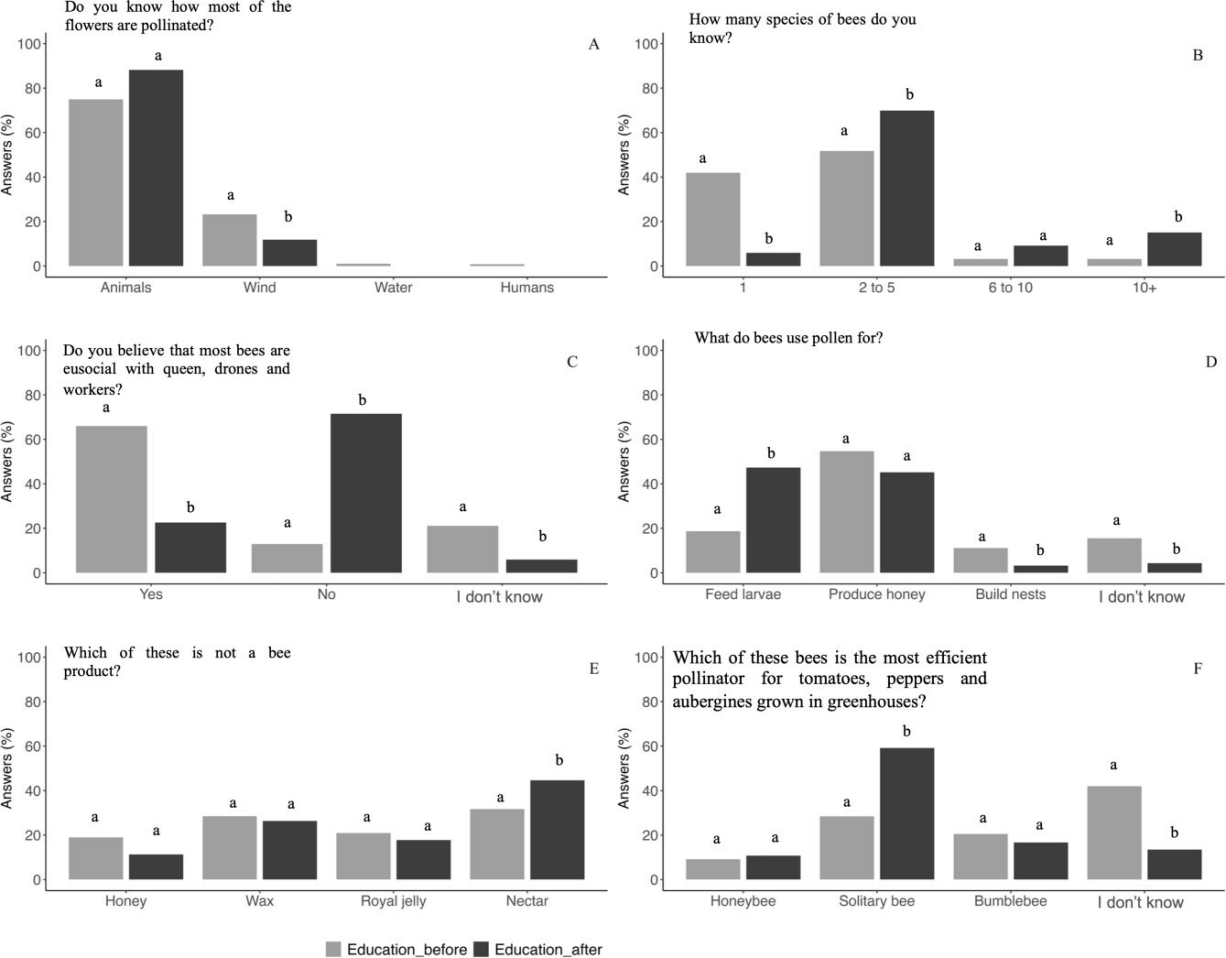
The evolution of the pollination knowledge in the preservice primary teachers at the University of Sevilla (pre- versus posttests comparison). The different letters among the columns mean a significant difference.

In general, the activities programmed for preservice primary teachers gave rise to an increment of the percentage of the students with a clear concept on the importance of pollination and the role of bees in this process (figure 6). In relation to the bee number knowledge, there was a trend toward a better knowledge of the high number of bee species as the percentage of the students recognizing only one bee species was drastically reduced from 41% to 5%, although the majority of the students only recognized between two and five pollinators (figure 6b). In relation to bee behavior, after the program, most of the preservice primary teachers (71%) were aware about the lack of eusocial character of some bee species, which constitutes a significant increase from the initial percentage of students being aware of that trait (13%; figure 6c, table S4).

Other aspects of bee biology were clear for most of the students before the training. This is the case for the previous ideas about honey production by bees. Before the training, most students had some basic understanding of the fact that not all bees produce honey, but after the training, there was a significant increase in the percentage of the students who correctly understood this concept, increasing from 67% to 88% (data not shown).

When we asked about the use of pollen by bees, it is noteworthy that initially more than 50% of the students thought that the pollen is destined to make honey, and less than 20% recognized that pollen is used by bees to feed their larvae. After the training, the percentage of the students knowing that pollen is used by bees to feed their larvae significantly increased to 47%. However, it is remarkable that 42% of the students still misunderstood the use of pollen by bees (figure 6d). Something similar occurred with the question about honeybee products, despite the positive impact of the training (from 31% to 44% of correct answers), many of the preservice primary teachers continued to believe that nectar is produced by honeybees (figure 6e).

### Perception about the importance of pollination of preservice primary teachers compared with the biology students

**Figure 7.**
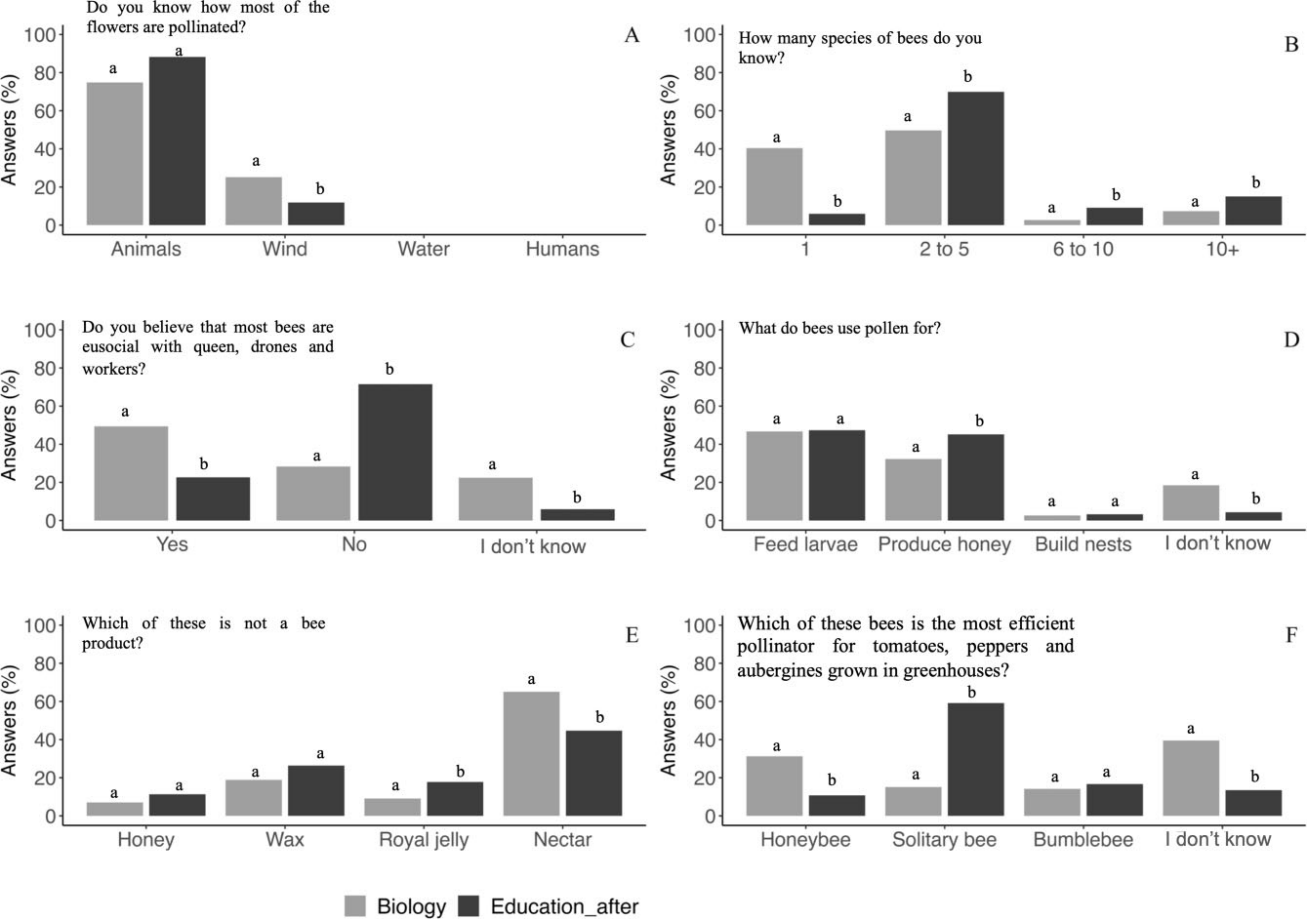
The evolution of the pollination knowledge in the preservice primary teachers. The results of these students after semester activities in class (pretests) are shown and compared with the results obtained by USevilla biology students. In each answer, the different letters between columns mean a significant difference between the degrees.

In general, the activities programmed for preservice primary teachers gave rise to an increment of the percentage of them with a clearer concept on the importance of pollination and the role of bees in this process when data from the posttest of these students were compared with the knowledge of the biology students (figure 7). In relation to the main pollinators, besides the percentage of the preservice primary teachers that acknowledged that the plants are pollinated mostly by animals was higher than that of biology students, the values were not significantly different (figure 7a). The percentage of the preservice primary teachers recognizing the existence of only one bee species was drastically low (5%) compared with the 40% of the biology students (figure 7b). Also, the percentage of the preservice primary teachers aware that more than 20,000 bee species exist (70%) was significantly higher than the percentage of the biology students (*χ*^2^(3) = 87.34, *p* < .0001; data not shown). In relation to bee behavior, after the program, most of preservice primary teachers (70%) were aware about the lack of eusocial character of some bee species, compared with 30% of the biology students (figure 7c).

Other aspects of bee life were clearer for most of the preservice primary teachers after the training, compared with the biology students. This is the case of the knowledge about the function of pollen in bee biology. After the training, there was a significant increase in the percentage of the preservice primary teachers who correctly understood this concept, in a percentage very similar to that of the biology students (around 50% for both; figure 7d).

When asked about the main pollinators in greenhouse crops, such as tomatoes, peppers, or eggplants (figure 7f), the percentage of the preservice primary teachers that gave the right answer was very similar to that of the biology students too (around 20%).

## Knowledge regarding the significance of bees as primary pollinators

In the realm of science education, prior research has delved into the significance of incorporating insects into elementary education curricula. Specifically, these studies were focused on addressing the fears and negative perceptions commonly associated with these creatures among students (Membiela et al. 2021, Puig and Gómez-Prado 2021, Membiela et al. 2023). Enhancing students’ attitudes toward insects is crucial for fostering meaningful learning experiences (Cho and Lee 2018, Sieg et al. 2018, Membiela et al. 2021, 2023, Puig and Gómez-Prado 2021). Moreover, motivation and positive attitudes are pivotal for promoting autonomous learning and cultivating cognitive and metacognitive competencies necessary for inquiry-based science instruction (Morón-Monge and Garcia-Carmona 2022). The aforementioned studies indicated that bees, unlike other insects, are particularly well regarded by students, and it is one of those topics that produces the least negative emotions (Reyes del Valle and Sánchez Torres 2020, Gómez-Prado et al. 2022, Membiela et al. 2023), although many students also consider bees dangerous because of a fear of stings (Schönfelder and Bogner 2017).

Taking into consideration the prevalent positive attitudes toward bees in society (Hall and Martins 2020) but also the ambivalent and negative associations (Vlasák-Drücker et al. 2022), as well as the limited research addressing students’ understanding of this topic at higher educational levels such as university, our study diverges from previous efforts by examining the depth of knowledge regarding the significance of bees as primary pollinators of both wild and cultivated plants among the three disciplines described (biology, agriculture, and preservice primary teachers) and between students from USevilla and UOuro. In addition, we explored the extent to which this knowledge evolves following an educational intervention with preservice primary teachers. Consequently, the main results are discussed in alignment with these dual objectives.

### Bee knowledge and bee decline

Although civic awareness of the seriousness of the problems facing the environment has generally increased in the last decades, misconceptions about the role of pollinators still remain, and only a few works have studied what general public know about pollination and pollinators (Golick et al. 2018, Jimenez et al. 2022). Although knowledge of certain concepts appeared consistent among students across disciplines, others demonstrated a pronounced reliance on their academic focus.

Most students exhibited a cohesive understanding of the concept of pollination, its significance for human well-being, and the pivotal role of bees as primary pollinators. Nonetheless, it is noteworthy that a minority of the students in our sample (approximately 20% of the preservice primary teachers and 8% of the agriculture students; data not shown) erroneously equated pollination with seed dispersal or germination. Other authors have also found that students often struggle to differentiate the pollination process from fertilization and seed dispersal within a plant's reproductive cycle (Golick et al. 2018, Lampert et al. 2019). These results suggest that students do not have a good understanding of the different phases of the reproductive cycle of plants. Moreover, students harbor misconceptions concerning the characteristics of pollinating organisms within ecosystems. Golick and colleagues (2018) also underscored the challenge students face in recognizing actionable steps for conserving pollinators, indicating a pressing need for more action-oriented pollinator conservation education. These challenging topics provide valuable insights for educators to tailor and refine their educational strategies to address these misconceptions (Jimenez et al. 2022).

Most of the students across all four disciplines (preservice primary teachers, agriculture students, and biology students at USevilla and biology students at UOuro) demonstrated a comparable level of knowledge regarding pollination vectors. This fundamental concept is typically covered during primary and secondary education, ensuring that all students enter university with a foundational understanding. However, variations in awareness of other aspects were clearly discipline dependent. For instance, a notable proportion of the agriculture students believed that wind serves as the primary pollination vector for crops, a viewpoint more prevalent in this discipline than in the others. This discrepancy can be attributed to the agriculture students’ familiarity with crops such as rice, corn, wheat, and various cereals, which are predominantly wind pollinated.

Many of the students encountered challenges in recognizing bumblebees as the primary pollinators in certain crops such as tomatoes, peppers, or eggplants, although this difficulty varied depending on their field of study. Particularly, the biology students at UOuro exhibited a higher degree of difficulty in this regard. Conversely, nearly 50% of the agriculture students identified bumblebees as the most significant pollinator for such greenhouse crops. This observation is unsurprising, considering that the choice of discipline reflects a heightened interest in crop production, and many students have backgrounds in family farming and are therefore familiar with the use of bumblebees in greenhouse settings (Andalusia is an eminently Spanish agricultural region). Moreover, the use of bees as pollinators in greenhouse crops is not a widespread practice in Brazil overall (Giannini et al. 2020).

An interesting finding in our study is the lack of knowledge of the students in any of the disciplines about bee diversity and bee biology. Therefore, the great majority of the students reported that they knew of the existence of up to five bees, and most of them believed that all the bees are eusocial, showing a clear knowledge bias toward the honeybee (*Apis mellifera*). That idea likely stems from the fact that the honeybee is one of the most widely grown species by humans to produce honey, a product well known to the general public. Therefore, because there are more than 20,000 bee species and because most bees are solitary, we can affirm that our students hold important misconceptions regarding bee diversity and their way of life. The same misconceptions have been reported for the general public, for whom the distinction between different types of bees seemed unimportant and tends to show a strong bias toward knowledge of honeybees (Vlasák-Drücker et al. 2022). In a similar line, when the students were asked about the use of pollen by bees, only the biology students, both in Spain and Brazil, presented a high percentage of correct answers, whereas a high proportion of the preservice primary teachers and of the agriculture students affirmed that pollen is used to make honey.

All these responses indicate that these students lack good knowledge of the life cycle of bees, where pollen is essential to feed the larvae and again clearly show a knowledge bias toward the honeybee. Although this bias has contributed to society's concern and awareness of pollinator decline, the limited perception of bee diversity may reduce efforts to conserve wild pollinator species (Geldmann and Gonzalez-Varo 2018, Vlasák-Drücker et al. 2022). Furthermore, although products derived from bee activities have significant benefits and profitable properties for humans because of their nutritional value and bioactive components (Hung et al. 2018, Lindao-Cordova et al. 2020, Durazzo et al. 2021), wild bees are fundamental for ecosystem maintenance (Constanza et al. 1997) and fruit production (Kleijn et al. 2015, Patel et al. 2021). Moreover, the conservation of honeybees often does not involve the conservation of other wild bee species, because of competition between them or disease transmission (Geldmann and González-Varo 2018). It is therefore necessary to focus on the knowledge and conservation of bee diversity, regardless of the benefits that humans derive from the particular species honeybee (Piccolo et al. 2022).

In our opinion, a greater knowledge of bees may contribute to higher willingness to support pollinator conservation and, as Aznar-Minguet and colleagues (2011) considered, the students’ apathy about educational and outreach aspects implies that innovative approaches need to be considered. According to Jimenez and colleagues (2022), implementing pollinator conservation efforts on university campuses requires extensive stakeholder buy-in because establishing habitat and teaching classes require dedicated time, space, finances, and personnel. In agreement with them, we think that the demand from the campus community needs to outweigh the potential costs to administrators if administrators are not inclined to support pollinator initiatives. As Colding and Barthel (2017) affirmed, higher educational institutions could play an excellent role in reconnecting people to the biosphere, and as many authors display, university students with knowledge of environmental problems could be leaders in the development of positive attitudes of society toward bee and pollinator decline (Zilahy and Huisingh 2009, Lozano et al. 2013).

Climate change stands out as the main cause of pollinator decline for most students rather than intensive agriculture and diseases. Although these aspects are important to both honeybees and native pollinators (Goulson et al. 2015, Wade et al. 2019), the lack of concern for factors other than climate change may be due to an absence of media coverage of these topics or may reflect the students’ inability to address perceived issues (Boehm 2012, Boehm and Singh 2013). In fact, the students may believe that eliminating the use of pesticides could result in a reduction in crop yields and could, consequently, have a negative impact on the agricultural sector and food prices. Indeed, this conflict is often reflected in the media because of protests by farmers, representing one of the decisions that governments frequently postpone because of its potential to generate social conflict.

As we have previously said, the knowledge of the students related to bee biology was very different among the disciplines. Most of the students were aware of the importance of pollinators to human society and have collected the information regarding bee collapsing from the media in general. According to 30% of the students, climate change is the most important cause of pollination decline. The pollinator crisis seems to have lost its impact on society because, nowadays, only the enormous problem of climate change is being argued (Althaus et al. 2021). In our opinion, the topic of climate change is shadowing other environmental issues, and this is being considered by our students the only problem that is affecting the planet globally. Moreover, as Sánchez-Bayo and Wyckhuys (2019) affirmed, unless we change our ways of producing food, insects as a whole will go down the path of extinction in a few decades, because the main cause of insect decline are habitat change and use of pesticides for our own benefit.

### Perception of future teachers about the importance of pollination

In overall terms, we can affirm that the educational intervention for preservice primary teachers was effective in understanding the importance of pollination, the role of bees as primary plant pollinators, the biology of bees and their role in honey production, and about bee diversity. However, in some of the questions, the preservice primary teachers still retained some previous ideas; a significant percentage of the students still had the incorrect idea that pollen is used to make honey, or they failed to correctly answer the question related to what a honeybee product is not. This observation suggests that the training course did not fully address or successfully dispel these misconceptions, probably because of the background of the students. Most of them have chosen social sciences during their schooling and they have spent little time learning natural sciences during their careers. That is the reason we think that additional education programs or outreach activities, such as the one that we have developed, are needed to fully convey these topics.

As Sotero and colleagues (2020) affirmed, teachers are an essential link of the process to improve the education programs that value scientific knowledge. They thought that students are sociocultural subjects that, when they are included in the school environment, bring with them knowledge, because of their experiences, so they should have initial and continuous training to foster enthusiastic learning in the children. Our students have actively participated in an open public conference; in addition, the students attended seminars given by bee experts, prepared posters that they defended in class, and created dissemination resources related to the importance of bee conservation; therefore, we have promoted project-based learning and learning from the action, improving in this way their knowledge about bees and pollination as an ecosystem service. From this methodological perspective focused on the active and critical role of the student toward socioenvironmental problems, it has allowed us to stimulate skills and attitudes necessary to act in society as an agent of change from an activism perspective (Reis 2020).

In our opinion, biology education could play a favorable role in behavior for sustainability. Students need to know much about bees to have a positive attitude toward them (Sieg et al. 2018). However, many of the primary preservice teachers, regrettably, lacked enthusiasm for experimental science. As we have previously mentioned, most of them came from a background in the social sciences, and as Davis and Palincsar (2021) asserted, elementary educators may not see themselves as science instructors. Interest in science can be fostered by employing student-centered approaches (Membiela et al. 2021, 2023), such as addressing pertinent topics such as the crisis facing bees and pollinators. As Sass and colleagues (2020) said, education for sustainable development is directed at supporting students in developing the necessary action competence in sustainable development. This is why we believe that outreach initiatives, where our students serve as both educators and learners, can cultivate a more positive attitude toward learning and teaching science, as well as to solve sustainable development issues.

### Perception about the importance of pollination of preservice primary teachers compared with that of biology students

The increase in the percentage of preservice primary teachers that, after the training, became aware of many aspects of bee biology and pollination was expected, as we have previously discussed. However, it was a great surprise to discover that, after the training, the level of correct answers of these students reached values similar to or higher than those of the biology students. It is important to notice that biology students have much more contact with concepts related to bee biology and pollination; therefore, we can affirm that the development of the educational training during the semester had succeeded.

**Figure 8.**
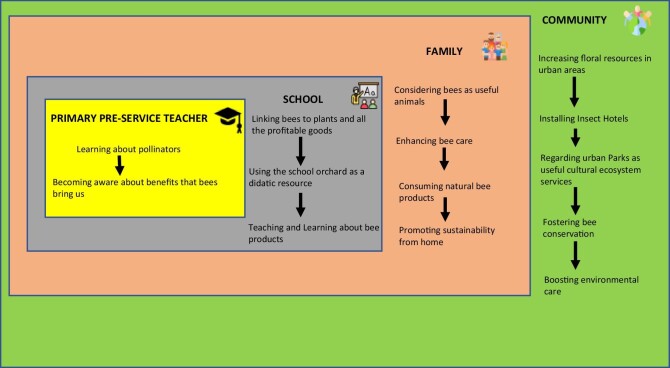
A multilevel framework for cascade effect. This figure was designed with Flaticon.com resources (www.flaticon.com).

As Ricoy and Sánchez-Martínez (2022) said, primary school education is exactly the right time to boost children's development of responsible behaviors toward protection of the environment; therefore, future teachers must be very well prepared in science to promote these environmental responsible behaviors in the children. Connecting school and community with science learning through real problems is a good strategy to enthuse children (Bouillion and Gómez 2001) and in agreement with Puig and Gómez-Prado (2021) and Morón-Monge and colleagues (2020), we think that we need to improve the scientific training of the preservice primary teachers to develop awareness and skills to help children (Hadjichambis and Reis 2020). At the same time, schools can recruit families to promote sustainability from home that, in turn, may imply improvements in the community from an environmental point of view. We show in figure 8 a possible multilevel framework to be considered from the faculty of education to the global community passing through the schools and the families. In this way, preservice primary teachers should be better educated in science in order to be excellent teachers that will be able to find creative ways to involve and engage their students in science learning, and the problem of bees and pollinators’ decline can be a good example to get planet awareness. Finally, we cannot forget that teaching can help to recognize environmental problems, to change the way that people think and act, and therefore, achieve a better and critical citizenship.

## Limitations and educational implications

The results of this study suggest some research challenges. Although the purpose of this study was not to establish generalizations, this work should be understood as a first approach that analyses student's prior knowledge of bee pollination and its decline. We focus this research on bees because of their crucial role as pollinators. However, future studies could be enriched by including other pollinator groups, which are also important for pollination and are significant for maintaining ecosystem health. Furthermore, it would be interesting to replicate the study by selecting another kind of representative sample of participants such as practicing teachers in order to identify the educational needs of teachers and therefore design coherent training proposals.

Garlick and Fallon (2023) promoted the ECO framework, which enhances three different ways of science engagement practice: formative engagement, codesign and coproduction, and broader outreach. In this way, and according to them, we can affirm that we have planned engagement formative activities related to scientific outreach to try to connect science and society through the future teachers and to try to get a better environmental citizenship that would use scientific research in decision-making.

Finally, as for its educational implications, this study tries to improve the transmission of science knowledge from preservice primary teachers to community through the learning of pollinators and its implications on the planet, as a very important ecosystem service; it can be useful to promote environmental cares, as well as scientific literacy. This is what we propose in our multilevel framework (figure 8). We firmly believe that, if we get that our students become aware of the benefits of bees, they will convey this knowledge among the children in the school, and their families will promote sustainability from home, and, consequently, communities will try to boost environmental care through different approaches in an urban global way.

## Supplementary Material

biae099_Supplemental_File

## Author Biography

Paula Daza (pdaza@us.es) is affiliated with the Universidad de Sevilla, Facultad de Biología/Facultad de Ciencias de la Educación, Departamento de Biología celular, in Sevilla, Spain. Montserrat Arista, Regina Berjano, and Pedro Ortiz are affiliated with the Universidad de Sevilla, Facultad de Biología, Departamento de Biología vegetal y Ecología, in Sevilla, Spain. Hortensia Morón-Monge is affiliated with the Facultad de Ciencias de la Educación, Departamento de Didáctica de las Ciencias experimentales y sociales, in Sevilla, Spain. Yasmine Antonini is affiliated with the ICEB-Universidade Federal de Ouro Preto, Departamento de Biodiversidade e Evolução, in Ouro Preto, Brazil.
