# Supplementary material for: Bee pollination and bee decline: A study about university students’ Knowledge and its educational implication
Source: Bioscience. 2024 Oct 26;74(12):851–66. doi: 10.1093/biosci/biae099 (PMC11660922; doi:10.1093/biosci/biae099)
Supplement: biae099_Supplemental_File [file biae099_supplemental_file.docx]

Supplementary TS1. Questionnaire applied to the students to measure the degree of knowledge regarding pollination in general in English and Spanish (original language) and Diagram of the validation process.

| **Q1.- Do you know what pollination is?**  1) Flower opening  2) Seed dispersal  3) Transfer of pollen to stigma  4) Seed germination |
| --- |
| **Q2.- How important do you think that pollination is for human well-being?**  1) A little  2) A lot  3) I don`t know |
| **Q3.-Do you know how most of the flowers are pollinated?**  1) By wind  2) By water  3) By animals  4) By humans |
| **Q4.- Which are the most efficient pollinators?**  1) Hummingbirds  2) Bats  3) Bees  4) Butterflies |
| **Q5.- How many species of bees do you know?**  1) Only one  2) Between 2 and 5  3) Between 6 and 10  4) More than 10 |
| **Q6.- How many species of bees do you think that exist?**  1) Around 50  2) Almost 500  3) More than 1000  4) More than 20000 |
| **Q7.- Do you believe that most bees are eusocial with queen, drones and workers?**  1) Yes  2) No  3) I don`t know |
| **Q8.- Do you believe that all bees produce honey?**  1) Yes  2) No  3) I don`t know |
| **Q9-What do bees use pollen for?**  1) To feed larvae  2) To produce honey  3) To build nests  4) I don´t know |
| **Q10.- Which of these is not a bee product?**  1) Royal jelly  2) Honey  3) Wax  4) Nectar |
| **Q11.- Which of these bees is the most efficient pollinator for tomatoes, peppers and aubergines grown in greenhouses?**  1) Honeybee  2) Solitary bee  3) Bumblebee  4) I don´t know |
| **Q12.- Which are the most efficient pollinators in an orchard?**  1) Honeybees  2) Solitary bees  3) Bumblebees  4) Humans |
| **Q13.- I have heard about pollinators` decline in:**  1) Biology lessons  2) Internet or television  3) On the street  4) I haven´t heard about it |
| **Q14.- Which of these elements will be in danger with the bees’decline?**  1) Oxygen  2) Algae  3) Humus  4) A third of animals and humans |
| **Q15.- Do you believe that solitary bees are better pollinators than honeybees?**  1) Yes  2) No  3) I don`t know |
| **Q16.- Rank in order of importance the main reasons for the disappearance of pollinators, with 1 being the most important reason...and 4 being the least important**  Modern agriculture  Climate change  Urbanization  Diseases |

| **Q1.- ¿Sabes qué es la polinización?**  1) La apertura de la flor  2) La dispersión de la semilla  3) La transferencia del polen al estigma  4) La germinación de la semilla |
| --- |
| **Q2.- ¿Cómo de importante es la polinización para el bienestar humano?**  1) Un poco  2) Mucho  3) No lo sé |
| **Q3.- ¿Sabes cómo son polinizadas la mayoría de las flores?**  1) Por el viento  2) Por el agua  3) Por los animales  4) Por las personas |
| **Q4.- ¿Quiénes son los polinizadores más eficientes?**  1) Los colibríes  2) Los murciélagos  3) Las abejas  4) Las mariposas |
| **Q5.- ¿Cuántas especies de Abejas conoces?**  1) Solo 1  2) Entre 2 y 5  3) Ente 6 y 10  4) Más de 10 |
| **Q6.- ¿Cuántas especies de Abejas crees que hay?**  1) Unas 50  2) Casi 500  3) Más de 1000  4) Más de 20000 |
| **Q7. - ¿Crees que la mayoría de las Abejas son sociales com reina, zânganos y obreras?**  1) Sí  2) No  3) No lo sé |
| **Q8.- ¿Crees que todas las Abejas producen miel?**  1) Sí  2) No  3) No lo sé |
| **Q10.- ¿Cuál de estos productos no es fabricado por las Abejas??**  1) La jalea real  2) La miel  3) La cera  4) El néctar |
| **Q11. - ¿Cuáles de estas Abejas son las más eficiente polinizando tomates, pimientos y berenjenas en los invernaderos?**  1) Las abejas melíferas  2) Las abejas solitarias  3) Los abejorros  4) No lo sé |
| **Q12.- ¿Cuáles son los polinizadores más eficientes en un huerto?**  1) Las abejas melíferas  2) Las abejas solitarias  3) Los abejorros  4) Las personas |
| **Q13.- ¿Dónde has oído hablar del declive de los polinizadores?:**  1) En las clases de biología  2) En internet o la televisión  3) Por la calle  4) No he oído hablar del tema |
| **Q14.- ¿Cuál de estos elementos estaría en peligro si desapareciesen las abejas?**  1) El oxígeno  2) Las algas  3) El humus  4) Una tercera parte de los animales y las personas |
| **Q15.- ¿Crees que las Abejas solitarias son mejores polinizadoras que las abejas melíferas?**  1) Sí  2) No  3) No lo sé |
| **Q16.- Ordena en orden de importancia la principal razón para la desaparición de los polinizadores, siendo 1 la razón más importante...y 4 la menos**  La agricultura moderna  El cambio climático  La urbanización  Las enfermedades |

Panel of experts

2nd opinion round

Panel of experts and Literature

First draft

Second draft

Statistical analysis of the answers

Work Team

Panel of experts

1st opinion round

Pilot test

Final Questionnaire

Work Team

Supplementary TS2. Number of answers to the items, in each grade of students from University of Seville (USevilla) and Federal University of Ouro Preto (UOuro)

| Questions | Answers | Grades USevilla | | | Grade UOuro | |  |
| --- | --- | --- | --- | --- | --- | --- | --- |
|  |  | Agriculture | Biology | Education | | Biology | Total |
| Q3.-Do you know how most of the flowers are pollinated? | By wind | 113 | 377 | 30 | | 41 | 561 |
|  | By water | 38 | 117 | 19 | | 5 | 179 |
|  | By animals | 0 | 5 | 0 | | 0 | 5 |
|  | By humans | 0 | 4 | 0 | | 0 | 4 |
| Q5-How many species of bees do you know | 1 | 12 | 61 | 211 | | 3 | 287 |
|  | 2 to 5 | 36 | 75 | 260 | | 35 | 406 |
|  | 6 to 10 | 1 | 4 | 16 | | 6 | 27 |
|  | 10+ | 0 | 11 | 16 | | 5 | 32 |
| Q7-Do you believe that most bees are eusocial with queen, drones and workers? | I don't know | 6 | 34 | 106 | | 11 | 157 |
|  | Yes | 33 | 75 | 332 | | 2 | 442 |
|  | No | 10 | 43 | 65 | | 34 | 152 |
| Q9-What do bees use pollen for? | To Feed larvae | 16 | 71 | 94 | | 23 | 204 |
|  | To produce honey | 27 | 49 | 275 | | 14 | 365 |
|  | To build nests | 3 | 4 | 56 | | 2 | 65 |
|  | I don't know | 3 | 28 | 78 | | 7 | 116 |
| Q10-Which of these is not a bee product? | Honey | 8 | 10 | 95 | | 3 | 116 |
|  | Wax | 10 | 27 | 143 | | 5 | 185 |
|  | Royal jelly | 10 | 13 | 105 | | 9 | 137 |
|  | Nectar | 21 | 93 | 159 | | 29 | 302 |
| Q11- Which of these bees is the most efficient pollinator for tomatoes, peppers and aubergines grown in greenhouses? | Honeybee | 5 | 64 | 46 | | 7 | 122 |
|  | Solitary bee | 8 | 31 | 143 | | 8 | 190 |
|  | Bumblebee | 22 | 29 | 103 | | 5 | 159 |
|  | I don't know | 14 | 81 | 211 | | 26 | 332 |

Supplementary TS3. General results of the chi square test comparing the answers of the students of Agriculture, Biology, Education, of the University of Seville for the six selected questions. Pre-test Preservice teachers were compared to the biology and agriculture students.

| Question | Factor | χ2 | df | P value |
| --- | --- | --- | --- | --- |
| Q3.-Do you know how most of the flowers are pollinated? | By wind | 90.11 | 6 | NS |
|  | By water |  |  |  |
|  | By animals |  |  |  |
|  | By humans |  |  |  |
| Q5-How many species of bees do you know | 1 | 15.117 | 6 | p=0.01 |
|  | 2 to 5 |  |  |  |
|  | 6 to 10 |  |  |  |
|  | 10+ |  |  |  |
| Q7-Do you believe that most bees are eusocial with queen, drones and workers? | Yes | 24.172 | 5 | p<0.001 |
|  | No |  |  |  |
|  | I don't know |  |  |  |
| Q09-What do bees use pollen for? | To feed larvae | 62.232 | 6 | p<0.001 |
|  | To produce honey |  |  |  |
|  | To build nests |  |  |  |
|  | I don't know |  |  |  |
| Q10-Which of these is not a bee product? | Honey | 54.515 | 6 | p<0.001 |
|  | Wax |  |  |  |
|  | Royal jelly |  |  |  |
|  | Nectar |  |  |  |
| Q11- Which of these bees is the most efficient pollinator for tomatoes, peppers and aubergines grown in greenhouses? | Honeybee | 80.029 | 6 | p<0.001 |
|  | Solitary bee |  |  |  |
|  | Bumblebee |  |  |  |
|  | I don't know |  |  |  |

Supplementary TS4. General results of the chi square test for the evolution of the pollination knowledge in the pre-service primary teachers of the University of Seville. This is a pre-post test comparison of only Preservice education teachers.

| Question | Factor | χ2 | df | p value |
| --- | --- | --- | --- | --- |
| Q3.-Do you know how most of the flowers are pollinated? | By wind | 15.14 | 5 | 0.001 |
|  | By water |  |  |  |
|  | By animals |  |  |  |
|  | By humans |  |  |  |
| Q5-How many species of bees do you know | 1 | 102.71 | 4 | p<0.001 |
|  | 2 to 5 |  |  |  |
|  | 6 to 10 |  |  |  |
|  | 10+ |  |  |  |
| Q7-Do you believe that most bees are eusocial with queen, drones and workers? | Yes | 227.71 | 4 | p<0.001 |
|  | No |  |  |  |
|  | I don't know |  |  |  |
|  |  |  |  |  |
| Q9-What do bees use pollen for? | To feed larvae | 67.51 | 5 | P<0.001 |
|  | To produce honey |  |  |  |
|  | To build nests |  |  |  |
|  | I don't know |  |  |  |
| Q10-Which of these is not a bee product? | Honey | 12.06 | 5 | p<0.005 |
|  | Wax |  |  |  |
|  | Royal jelly |  |  |  |
|  | Nectar |  |  |  |
| Q11- Which of these bees is the most efficient pollinator for tomatoes, peppers and aubergines grown in greenhouses? | Honeybee | 68.473 | 5 | p<0.001 |
|  | Solitary bee |  |  |  |
|  | Bumblebee |  |  |  |
|  | I don't know |  |  |  |
